# Limitations of PCR detection of filarial DNA in human stools from subjects non-infected with soil-transmitted helminths

**DOI:** 10.1051/parasite/2021046

**Published:** 2021-05-27

**Authors:** Maxime P. M. Doret, Hugues C. Nana-Djeunga, Narcisse Nzune-Toche, Sébastien D. S. Pion, Cédric B. Chesnais, Michel Boussinesq, Joseph Kamgno, Emmanuelle Varlet-Marie, Sabrina Locatelli

**Affiliations:** 1 Institut de Recherche pour le Développement (IRD), UMI 233-INSERM U1175-University of Montpellier 34394 Montpellier Cedex 5 France; 2 Centre for Research on Filariasis and Other Tropical Diseases (CRFilMT) PO Box 5797 Yaoundé Cameroon; 3 Department of Public Health, Faculty of Medicine and Biomedical Sciences (FMBS) PO Box 1364 Yaoundé Cameroon; 4 Department of Parasitology–Mycology, Centre Hospitalier Universitaire (CHU) of Montpellier, University of Montpellier 34295 Montpellier Cedex 5 France

**Keywords:** *Mansonella perstans*, *Loa loa*, Stool sample, PCR, Cameroon

## Abstract

The standard techniques for diagnosis of human filariasis are the microscopic examination of blood smears or skin biopsies, which are relatively invasive and poorly sensitive at low levels of infection. Recently, filarial DNA has been detected in fecal samples from non-human primates in Central Africa. The aim of this study was to demonstrate proof-of-concept of a non-invasive molecular diagnosis technique for human filariasis by targeting fragments of 12S rDNA, Cox1, ITS1 and LL20-15kDa ladder antigen-gene by conventional PCR in DNA extracted from stool samples of 52 people infected with *Mansonella perstans* and/or *Loa loa*. Of these, 10 patients were infected with soil-transmitted helminths (*Trichuris trichiura* and/or *Ascaris lumbricoides*), and none were positive for *Necator americanus*. Interestingly, no filarial gene fragments were detected in the stools of any of the 52 patients. Future studies should evaluate whether a co-infection with soil-transmitted helminths causing gastrointestinal bleeding and likely allowing (micro)filaria exit into the digestive tract, may facilitate the molecular detection of filarial DNA fragments in stool samples.

## Abstract (fr)

Les techniques standards de diagnostic des filarioses humaines (examen microscopique de gouttes épaisses ou de biopsies cutanées) sont relativement invasives et peu sensibles à de faibles niveaux d’infection. De l’ADN de filaires a été récemment détecté dans des échantillons de fèces de primates non-humains en Afrique centrale. L’objectif de cette étude était de démontrer la preuve de concept d’un diagnostic moléculaire non invasif des filarioses chez l’homme en ciblant des fragments d’ADNr 12S, Cox1, ITS1 et l’antigène LL20-15kDa par PCR classique. L’ADN a été extrait d’échantillons de selles de 52 personnes infectées par *Mansonella perstans* et/ou *Loa loa*. Parmi ces patients, dix étaient infectés par des géohelminthes (*Trichuris trichiura* et/ou *Ascaris lumbricoides*) et aucun n’était positif pour *Necator americanus*. De manière intéressante, aucun fragment de gène de filaires n’a été détecté dans les selles des 52 patients. Des études futures devraient être menées pour évaluer si une coinfection avec des géohelminthes (provoquant des hémorragies gastro-intestinales et permettant probablement l’effraction de (micro)filaires dans le tube digestif) facilite la détection moléculaire de fragments d’ADN de filaires dans les selles.

## Introduction

Filariases are vector-borne infections caused by nematode parasites. Adults of the main species infecting humans live in subcutaneous or deep nodules (*Onchocerca volvulus*), in fascia layers (*Loa loa*), in the lymphatic circulation (lymphatic filariae), or in body cavities (*Mansonella perstans*, *M. ozzardi*). Female worms produce embryos called microfilariae (mf) that migrate into the dermis (*O. volvulus*) or into the bloodstream (*L. loa*, lymphatic filariae, *M. perstans*). Diagnosis relies on the detection of mf in skin biopsies for onchocerciasis or in a calibrated thick blood smear (CTBS) for the other filariases. These techniques are relatively invasive, and their sensitivity decreases with lower infection intensity. Methods using a molecular approach, like polymerase chain reaction (PCR) [5] and loop-mediated isothermal amplification (LAMP) [10, 12], are more specific and sensitive. Recently, *Mansonella* sp. DNA was detected in fecal samples from non-human primates (NHP) [6]. This study aimed to demonstrate the proof-of-concept of non-invasive diagnosis of human filariasis in stool samples using a conventional PCR method, and to determine whether filarial DNA detection is associated with co-infections with soil-transmitted helminths (STH) causing blood loss while feeding on the host intestinal mucosa.

## Materials and methods

### Ethics guidelines

The study, conducted in Cameroon, was approved by the Centre Regional Ethics Committee for Human Health Research (No. 00837/CRERSCH/2019). All volunteers were informed one week before the beginning of the study and signed a consent form prior to enrollment.

### Study site, samples collection and microscopic analyses

The subjects included in this study (aged 13–88 years) participated in a project conducted in 2015 in 92 villages of the Okola Health District (Centre Region) to evaluate the efficacy of a rapid diagnostic tool called LoaScope to identify subjects with high *L. loa* microfilarial densities (MFD) [9]. During this “Test-and-not-Treat” (TaNT) project, individuals with ≥20,000 *L. loa* mf/mL of blood, who were at risk of developing a serious reaction after ivermectin treatment, did not receive the drug. In 2018, 52 of these excluded individuals with high *L. loa* and/or *M. perstans* MFD participated in the present study. During the information phase of the study, patients were provided with a 60 mL vial for stool collection and were instructed to hand it with fresh stool (produced the morning of the visit) to the field team the day of the capillary blood collection. Capillary blood samples were collected between 10 am and 4 pm (time of highest *L. loa* MFD in the peripheral circulation). *Loa loa* and *M. perstans* MFD were measured by CTBS. Upon receipt of the stool sample, 2 mL (about 1 g of stool) were put in a 15 mL tube and 2 mL of RNA*later*^®^ (Ambion, Austin, TX, USA) were added. Both tubes were transferred to the CRFilMT laboratory in Yaoundé in a cooler with icepacks. The samples in RNA*later*^®^ were stored at −20 °C for further molecular analyses. For the intestinal parasite eggs counts, Kato-Katz analyses were performed the day after collection on a small amount of stool (41.7 mg template) taken directly from the 60 mL tube, after homogenization. The remaining stool material was resuspended in formalin for the additional detection of gastrointestinal parasites using the Mini-FLOTAC technique.

### DNA extraction

Fecal DNA was extracted using a QIAamp Fast Stool DNA Mini kit (Qiagen, Valencia, CA, USA), following the manufacturer’s instructions. Briefly, 1.5 mL of fecal-RNAlater^®^ mixture was re-suspended in stool lysis buffer and clarified by centrifugation. The supernatants were treated with an InhibitEx buffer, subjected to proteinase K digestion, and passed through a DNA binding column. Bound DNA was eluted in 100 μL elution buffer. To investigate the presence of gastro-intestinal parasites, a modified DNA extraction protocol was adopted, after the clarification. This step consisted of additional homogenization in tubes containing silica beads of different size (lysis matrix E) in a FastPrep-24 mill (MP Biomedical, Eschwege, Germany) and overnight incubation at 56 °C to better expose the parasite DNA in eggs. Dried blood spots (DBS) from 20 additional pharmacologically untreated individuals with high MFD (10 infected with *L. loa* only (110,360–269,520 mf/mL) and 10 with *M. perstans* only (960–4020 mf/mL)) enrolled in the project mentioned above were selected as positive controls, and DNA extraction was performed using a NucliSENS^®^ kit (bioMérieux, France) [11]. Data on mf/mL from the 20 individuals are available from the corresponding author on reasonable request.

### Diagnosis of filarial and STH DNA from stool samples

To assess the efficacy of the DNA extraction, we ran a PCR targeting a 460–500-bp mitochondrial DNA fragment spanning the human 12S rDNA region. Subsequently, to detect the presence of filariae, we ran nested PCRs targeting fragments of two mitochondrial genes (12S rDNA and Cox1), one-step PCRs targeting fragments of the Internal Transcribed Spacer 1 (ITS1), and the LL20 15KDa ladder antigen gene (see Table 1). PCRs were run for DNA extracted from DBS from 20 individuals, for DNA extracted from stools from 52 additional individuals alone, and for DNA extracted from stools from the latter individuals with an addition of 0.1 μL of *M. perstans* DNA and 0.05 μL of *L. loa* DNA extracted from the DBS positive samples to check for the presence of PCR inhibitors. Negative controls were included to check for contaminations. To validate this protocol in fecal samples, PCRs targeting a fragment of the ITS1 (using primers Mp-Sen-F and Mp-Sen-R) were also run on six DNA samples extracted from chimpanzees positive for *Mansonella* sp. (for 12S rDNA or Cox1) [6]. To confirm the results of the stool microscopic examinations, nested or semi-nested PCRs targeting ITS1 or ITS2 fragments of the STH genomes, respectively, were also run (primers, PCR conditions and references listed in Table 1). The sequences were assembled and corrected manually with SeqMan DNAstar (Laser-Gene, DNASTAR, Inc., Madison, WI, USA), then compared to the reference sequences available in GenBank, using the Nucleotide Basic Local Alignment Search Tool (BLASTn).

**Table 1:** Primers list, PCR conditions, and references.

Host target	Gene	Primer			Thermal profile^a^							Ref.
		Designation	Sequence (5′ – 3′)	Product	Step 1		Step 2	Step 3			*N*	
				Size (bp)	*T*	*D*	*T*	*D*	*T*	*D*		
*Homo sapiens*	HVRI	L15997	CACCATTAGCACCCAAAGCT	~400	94	30	50	30	72	30	40	[17]
		H16498	CCTGAAGTAGGAACCAGATG									
*L. loa*/*M. perstans*	*12S rDNA*	12SdegF2/	ATTACYTATTYTTAGTTTA	~600	94	30	45	30	72	45	40	[2]
		12SnemR2	CTACCATACTACAACTTACGC									
		12SF/	GTTCCAGAATAATCGGCTA	~450	94	30	54	30	72	30	35	
		12SdegR	ATTGACGGATGRTTTGTACC									
*L. loa*/*M. perstans*	*coxI*	FCo1extdF1	TATAATTCTGTTYTDACTA	~970	94	30	44	30	72	60	40	[2]
		FCo1extdR1	ATGAAAATGAGCYACWACATAA									
		COIintF/	TGA TTG GTG GTT TTG GTA A	~650	94	30	45	30	72	45	35	
		COIintR	ATA AGT ACG AGT ATC AAT ATC									
*Oesophagostomum*/*Necator* sp.	*ITS2*	NC1	ACGTCTGGTTCAGGGTTGTT	NA	94	30	50	30	72	45	45	[7]
		NC2	TTAGTTTCTTTTCCTCCGCT									
		OesophITS2	TGTRACACTGTTTGTC-GAAC	250-300	94	30	55	30	72	30	35	
		NC2	TTAGTTTCTTT-TCCTCCGCT									
*Thrichuris trichiura*	*ITS2*	ExtITS2	GGATCACTTGGCTGGTAG	NA	94	30	56	30	72	45	45	[8]
		NC2	TTAGTTTCTTTTCCTCCGCT									
		IntITS2	CTTGAATACTTTGAACGCACATTG	~700	94	30	49	30	72	45	35	
		NC2	TTAGTTTCTTTTCCTCCGCT									
*Acaris lumbricoides*	*ITS1*	ITS F1	CGAGCAGAAAAAAAAAAGTCTCC	NA	94	30	50	45	72	45	45	[3]
		ITS R1	GGAATGAACCCGATGGCGCAAT									
		ITS F2	CGAGCAGAAAAAAAAAAAAGTCTCC	~500	94	30	52	30	72	30	35	
		ITS R2	GCTGCGTTCTTCATCGAT									
*Mansonella perstans*	*ITS1*	Mp-SEN-F	AGGATCATTAACGAGCTTCC	~187	94	30	50	30	72	30	35^b^	[1]
		Mp-SEN-R	CGAATATCACCGTTAATTCAGT									
*Loa loa*	LL20 15KDa	15r3-LL-F	CGAAAAATTATAGGGGGAAAC	~148	94	30	50	30	72	30	35^b^	[15]
		15r3-LL-R	TCGTAGACCAAACTGCGAAC									

a All PCRs start at 95 °C – 15 min and finish at 72 °C – 10 min.

b 35× for DBS and 45× for stool samples.

NA: not available. In all cases, the PCR reaction was performed in 50 μL reaction volume containing 10 μL and 5 μL of template DNA for primary and nested PCR respectively, 10 pM of each primer, 25 μL HotStarTaq Master Mix (Qiagen, Courtaboeuf, France), providing a final concentration of 1.5 mM MgCl_2_ and 200 μM each dNTP, 1 μg of Bovine Serum Albumin (SIGMA, USA).

*Abbreviations*: Step 1, denaturation; Step 2, annealing; Step 3, elongation; *T*, temperature (°C); *D*, duration (s); *N*, number of cycles.

## Results and discussion

### Detection of *M. perstans* and *L. loa*

Six DNA samples extracted from chimpanzee feces were positive for *Mansonella* sp., using primers MP-Sen targeting the ITS1, confirming previous results [6]. Although the detection of human DNA is not a guarantee that pathogen DNA has not been degraded, it was successfully amplified in all 52 stool samples. An RT-qPCR inhibition screening test was conducted on a subset of samples by targeting the human albumin gene. No PCR method was able to amplify *M. perstans* and *L. loa* target fragments from the DNA extracted. However, samples in which a dilution of DNA extracted from DBS was added were all positive. The same DNA extraction methods were applied for the chimpanzee fecal samples for which we were able to detect the presence of filariae.

### Detection of soil-transmitted helminths

All samples were analyzed for the presence of *Necator americanus*, *Trichuris trichiura*, and *Ascaris lumbricoides* by the Kato-Katz technique, Mini-FLOTAC and conventional PCR (Table 2). Five participants were infected with *T. trichiura*, four with *A. lumbricoides*, one with both species, but none with *N. americanus*. Thus, altogether, the number of patients infected with STH was low. These findings corroborate those obtained during a National survey conducted in 2010 [14], which reported low prevalence of infection in school children living in the Centre Region (10.5, 18.6 and 2.7% for *A. lumbricoides*, *T. trichiura* and hookworms, respectively), and those obtained in 2020 in adults living in villages included in the TaNT project mentioned above (prevalence of 1.4% for *A. lumbricoides* and 2.2% for *T. trichiura*) (Nana-Djeunga, unpublished results). This situation probably results from the fact that (a) the health areas neighboring the TaNT villages have been treated with ivermectin for about two decades to fight onchocerciasis; (b) most of the people living in the TaNT villages benefitted from annual treatment with ivermectin between 2015 and 2018 (subjects excluded from this treatment represent less than 3% of those tested with the LoaScope); and (c) school-aged children of the whole health district have received regular mebendazole treatment since 2007. Of note, it has been shown recently that, in areas where ivermectin is administered as a mass treatment, the impact is greater on STH transmission compared to where only mebendazole has been administered [4]. Given the absence of hookworm infection, the low proportion of participants infected with *T. trichiura* or *A. lumbricoides*, and the low egg counts recorded (Table 2), STH-related blood loss in the intestine was probably minimal. This may explain the absence of DNA from blood-dwelling mf in the samples examined. Importantly, among the six chimpanzee stool samples that we found to be positive for *Mansonella* sp., five were infected with *Oesophagostomum* sp. and/or *Necator* sp. [6].

**Table 2:** CTBS results for *L. loa* and *M. perstans* and Kato-Katz, Mini-FLOTAC and PCR results for soil-transmitted helminths.

No. stool sample	Giemsa staining *M. perstans* (mf/mL)	Giemsa staining of *L. loa* (mf/mL)	*T. trichiura* egg count/Kato-Katz (epg)	*T. trichiura* egg count/mini FLOTAC	PCR ITS2 *T. trichiura*	*A. lumbricoides* egg count/Kato-Katz (epg)	*A. lumbricoides* egg count/mini FLOTAC	PCR ITS1 *A. lumbricoides*
S-001	1340	57,140	0	0	NEG	48	0	NEG
S-002	240	35,260	0	0	NEG	0	20	NEG
S-003	0	29,280	0	0	NEG	0	0	NEG
S-004	0	65,740	0	0	NEG	0	0	NEG
S-005	0	71,500	0	0	NEG	0	0	NEG
S-006	0	47,380	0	0	NEG	0	0	NEG
S-007	0	18,840	0	0	NEG	0	0	NEG
S-008	0	23,200	0	0	NEG	0	0	NEG
S-009	0	92,000	0	0	NEG	0	0	NEG
S-010	0	51,040	0	15	**POS**	0	0	NEG
S-011	0	58,640	0	0	NEG	0	0	NEG
S-012	0	17,260	0	0	NEG	0	0	NEG
S-013	20	3300	0	0	NEG	0	0	NEG
S-014	0	35,780	0	0	NEG	0	0	NEG
S-015	0	223,700	0	0	NEG	0	0	NEG
S-016	80	46,760	0	0	NEG	0	0	NEG
S-017	100	10,300	0	0	NEG	0	0	NEG
S-018	160	28,520	0	0	NEG	0	0	NEG
S-019	0	13,740	0	0	NEG	0	5	NEG
S-020	0	1960	0	5	NEG	0	0	NEG
S-021	0	29,200	0	0	NEG	0	0	NEG
S-022	0	45,560	0	0	NEG	0	0	NEG
S-023	0	137,180	ND	ND	NEG	ND	ND	NEG
S-024	660	24,920	0	0	NEG	0	0	NEG
S-025	140	20,180	0	0	NEG	0	0	NEG
S-026	0	19,060	0	0	NEG	0	0	NEG
S-027	0	4480	0	0	NEG	0	0	NEG
S-028	0	41,380	0	0	NEG	0	0	NEG
S-029	0	5000	0	0	NEG	0	0	NEG
S-030	0	66,400	0	0	NEG	0	0	NEG
S-031	200	25,640	0	0	NEG	0	0	NEG
S-032	20	46,720	0	0	NEG	0	0	NEG
S-033	420	41,180	24	15	NEG	8088	3700	**POS**
S-034	0	45,500	0	0	NEG	0	0	NEG
S-035	360	8600	ND	ND	NEG	ND	ND	NEG
S-037	240	7500	0	ND	NEG	0	ND	NEG
S-038	0	22,900	0	0	NEG	0	0	NEG
S-039	0	8840	0	0	NEG	0	0	NEG
S-040	0	8000	0	5	NEG	0	0	NEG
S-041	0	18,700	0	0	NEG	0	0	NEG
S-042	0	27,760	0	0	NEG	0	0	NEG
S-043	140	72,920	0	0	NEG	0	0	NEG
S-044	0	51,820	0	0	NEG	0	0	NEG
S-045	0	1800	0	0	NEG	0	0	NEG
S-046	0	25,680	0	0	NEG	0	5	NEG
S-047	0	72,500	0	0	ND	0	0	NEG
S-048	0	2960	0	0	NEG	0	0	NEG
S-049	0	30,600	0	5	NEG	0	0	NEG
S-050	0	ND	0	0	NEG	0	0	NEG
S-051	ND	ND	0	5	NEG	0	0	NEG
S-052	ND	ND	0	0	NEG	0	0	NEG

(1) PCR 12S rDNA (human mitochondrial DNA): all positive. (2) PCR 12S rDNA (filariae): all negative. (3) PCR cox1 (filariae): all negative. (4) PCR ITS1 *M. perstans:* all negative. (5) PCR ITS1 *M. perstans-*inhibition test (0.1 μL DBS DNA): all positivePCR *L. loa* LL20-15kDA: all negative. (6) PCR *L. loa* LL20-15kDA-inhibition test (0.05 μL DBS DNA): all positive. (7) Hookworm egg count/Kato-Katz (epg)/mini FLOTAC/PCR ITS2 *N. americanus/Oesophagostomum* spp. (8) ND: not determined.

## Conclusion

We obtained PCR-negative results for the detection of filarial DNA in human feces from infected patients. RT-qPCR may display higher sensitivity compared to conventional PCR, and this may be the limitation of this study. However, filarial DNA detection appears to work better in NHPs co-infected with intestinal parasites. If co-infection with intestinal parasites is necessary, then the present study clearly suffered from a lack of statistical power. We had assumed that a significant proportion of subjects would be infected with STH, but this was not the case. Further studies should target individuals co-infected with STH (especially hookworms) and filariae to test our hypothesis. It should be noted that gastrointestinal bleeding may also be caused by other diseases such as colon cancer, hemorrhoids or ulcerations, and therefore a fecal occult blood test could complement the search for STH. In addition, exploring the presence in fecal samples of cell-free DNA (cfDNA) fragments from various pathogens is an approach worth investigating in the future [18]. The development of a reliable, non-invasive test combining the detection of different parasites in one sample could be useful when samples are collected in remote regions with difficult access to points of care [13, 16].

## Availability of data and materials

Datasets and questionnaires are available from the corresponding author under reasonable request.

## Authors’ contributions

Study design: HCND, SDSP, CBC, SL. Data collection and microscopic analyses: HCND, NNT. PCR experiments: MPMD, SL. Data analysis: MPMD, HCND, SL. Writing: MPMD, SL with input from all authors. All authors read and approved the final manuscript.

## Conflict of interest

The authors declare that they have no conflict of interest.
